# TANGO2 deficiency disease is predominantly caused by a lipid imbalance

**DOI:** 10.1242/dmm.050662

**Published:** 2024-06-05

**Authors:** Michael Sacher, Jay DeLoriea, Mahsa Mehranfar, Cody Casey, Aaliya Naaz, Chiara Gamberi

**Affiliations:** ^1^ Department of Biology, Concordia University, Montreal H4B 1R6, Canada; ^2^ Department of Anatomy and Cell Biology, McGill University, Montreal H3A 0C7, Canada; ^3^ Department of Biology, Coastal Carolina University, Conway, SC 29526, USA; ^4^ Department of Chemistry and Biochemistry, Concordia University, Montreal H4B 1R6, Canada

**Keywords:** TANGO2 deficiency disease, Lipid imbalance, Metabolic crises, Neurodevelopmental disease

## Abstract

TANGO2 deficiency disease (TDD) is a rare genetic disorder estimated to affect ∼8000 individuals worldwide. It causes neurodegeneration often accompanied by potentially lethal metabolic crises that are triggered by diet or illness. Recent work has demonstrated distinct lipid imbalances in multiple model systems either depleted for or devoid of the TANGO2 protein, including human cells, fruit flies and zebrafish. Importantly, vitamin B5 supplementation has been shown to rescue TANGO2 deficiency-associated defects in flies and human cells. The notion that vitamin B5 is needed for synthesis of the lipid precursor coenzyme A (CoA) corroborates the hypothesis that key aspects of TDD pathology may be caused by lipid imbalance. A natural history study of 73 individuals with TDD reported that either multivitamin or vitamin B complex supplementation prevented the metabolic crises, suggesting this as a potentially life-saving treatment. Although recently published work supports this notion, much remains unknown about TANGO2 function, the pathological mechanism of TDD and the possible downsides of sustained vitamin supplementation in children and young adults. In this Perspective, we discuss these recent findings and highlight areas for immediate scientific attention.

## Teaser

**Summary:** TANGO2 deficiency disease, a rare condition featuring neurodegeneration and life-threatening metabolic crises, has a signature of lipid imbalance that can be compensated by supplementation of the CoA- and lipid-precursor vitamin B5.

## Introduction

**Fig. 1.**
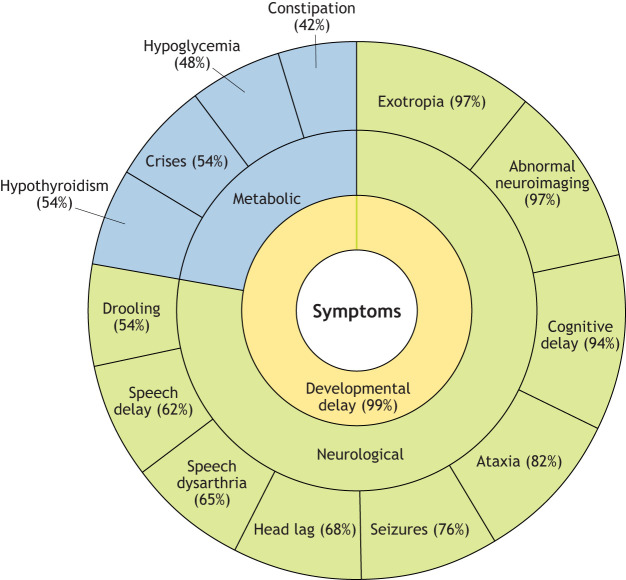
**Features and phenotypic variation of TANGO2 deficiency disease.** A collection of neurodevelopmental, muscular and systemic defects, including developmental delay, life-threatening metabolic crises and cardiac arrhythmias typically appear in late infancy in TANGO2 deficiency disease (Berat et al., 2021; Dines et al., 2019; Hoebeke et al., 2021; Mingirulli et al., 2020; Miyake et al., 2022; Schymick et al., 2022). The severity varies with the mutation type and its mono- or bi-allelic status, and it also varies within individuals of the same family (Miyake et al., 2022; Schymick et al., 2022), suggesting the contribution of other factors. Percentages indicating the prevalence among patients with TDD are shown.

Bi-allelic loss-of-function mutations in the transport and Golgi organization homolog 2 (*TANGO2*) gene (NCBI gene ID: 128989) are linked to a rare neurometabolic disorder known as TANGO2 deficiency disease (TDD, OMIM #616878). TDD is associated with significant morbidity due to cardiac arrhythmias that happen during episodic metabolic decompensation (Fig. 1). Typically, delays in development, speech and cognition, as well as dystonia (muscle spasms and twisting), seizures and hypothyroidism characterize TDD (Kremer et al., 2016; Lalani et al., 2016; Miyake et al., 2023). Metabolic crises cause muscle weakness and rhabdomyolysis, cardiac arrythmia, ataxia and slurred speech (dysarthria), and may result in hypoglycemia and encephalopathy (Fig. 1).

**Fig. 2.**
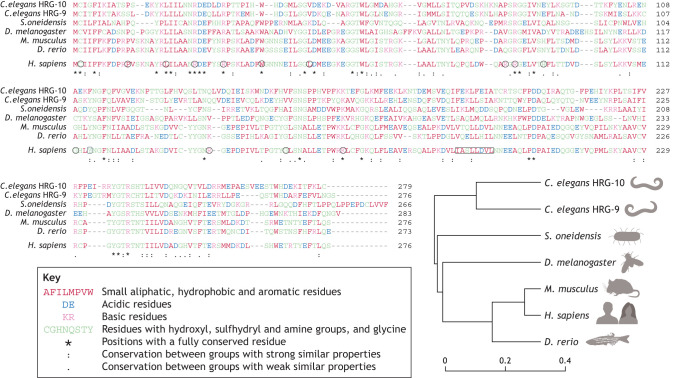
**TANGO2 homologs.** Multiple sequence alignment of TANGO2-like proteins from the worm *Caenorhabditis elegans* (HRG-9 and HRG-10), the bacterium *Shewanella oneidensis*, the fruit fly *Drosophila melanogaster*, mice (*Mus musculus*), humans (*Homo sapiens*) and zebrafish (*Danio rerio*). GenBank sequences were downloaded in FASTA format, aligned with Clustal Omega (Madeira et al., 2022), and manually edited and annotated. Note that Clustal Omega orders sequences according to similarity scores. For easier comparison, the human sequence has been extracted and reported below with further annotations. The adjacent taxonomy tree illustrates sequence relatedness (arbitrary units used to compare branching architectures within a guided tree). Residue color coding and symbols indicating residue conservation are indicated. In the human sequence, tyrosine (Y) 116, implicated in heme binding in worms and bacteria, is highlighted (rounded square). This tyrosine is conserved in all the orthologs shown except for *C. elegans* HRG-10. Residues in human TANGO2 affected by pathological mutations are circled; the likely pathologic deletion of residues 190-197 is boxed. Variants of pathological significance were extracted from UniProt (UniProt, 2023). UniProt sequence identifiers: *C. elegans* HRG-9 (Q22009) and HRG-10 (Q9U1Q8), *S. oneidensis* (Q8EKG7), *D. melanogaster* (Q9VYA8), *M. musculus* (P54797), *H. sapiens* (Q6ICL3) and *D. rerio* (Q6AXK1).

First discovered in a screen for proteins involved in constitutive secretion and Golgi organization in a *Drosophila melanogaster* cell line (Bard et al., 2006), the function of the TANGO2 protein remains unclear. It is expressed in most human tissues (Fagerberg et al., 2014) and has been linked to several functions in intracellular trafficking, mitochondria (Berat et al., 2021; Heiman et al., 2022; Jennions et al., 2019; Milev et al., 2021) and, potentially, heme transport (Sun et al., 2022). TANGO2 is evolutionarily conserved from humans to invertebrates, including the key model organisms *Drosophila*, *Caenorhabditis elegans* and zebrafish (Fig. 2) that enable probing of the molecular and genetic basis of TDD. TANGO2-like proteins have also been found in prokaryotes (e.g. *Shewanella oneidensis*) (Han et al., 2023), suggesting an evolutionarily conserved function.

As the etiology of TDD and TANGO2 protein function are poorly understood, there is still no cure for TDD, yet discovery efforts are being made on both fronts.

As the etiology of TDD and TANGO2 protein function are poorly understood, there is still no cure for TDD, yet discovery efforts are being made on both fronts. A natural history study revealed that vitamin B complex or multivitamins ameliorated TDD symptoms (Miyake et al., 2023) and prevented metabolic crises and cardiac arrhythmias. Therefore, it is recommended to start supplementation of all eight B vitamins as early as possible upon diagnosis (Sandkuhler et al., 2023a). However, treatment duration and appropriate dosage remain undetermined. Moreover, it is unknown whether the remediating effect of vitamin B complex is due to one or a combination of several vitamins. Furthermore, excess of vitamin B6 can result in peripheral neuropathy, and its blood monitoring is recommended when taking a high dosage of vitamin B complex (Miyake et al., 2018). Given the need for prolonged vitamin B complex supplementation and the potential for acute and chronic hypervitaminosis resulting from excessive B vitamin intake (Hanna et al., 2022), determining the optimal treatment plan is essential.

## Vitamin B treatment indicates that lipid homeostasis defects underlie TDD

To optimize treatment plans for patients with TDD, several studies have further assessed the effect of individual B vitamins in patients and disease models. For example, in a recent preprint, vitamin B9 (folate) treatment of cardiomyocyte cell lines generated from patient-derived induced pluripotent stem cells (Zhang et al., 2022 preprint) notably decreased premature ventricular contractions within 4-12 h of treatment. This did not have any significant effect on corrected QT interval (QTc) prolongation, which is a heart rate measurement used to detect arrythmia.

A recent case report (Yilmaz-Gumus et al., 2023) found a noticeable improvement in TANGO2 deficiency-related metabolic crisis, especially in mental status and rhabdomyolysis, within 24 h of administration of a combination of vitamin B5 (pantothenic acid) and vitamin B3 (niacin) in a patient with the *TANGO2* homozygous pathogenic variant c.380+1G>A. In a recent preprint, only vitamin B5 was administered to a patient with TDD and significant improvement was noted almost immediately, which persisted over several years (Li et al., 2023 preprint). Asadi et al. (2023) reported that vitamin B5 restored several defects associated with a *Tango2* loss-of-function mutation in *Drosophila* and improved membrane trafficking defects in *TANGO2* knockout human fibroblasts in a time-dependent manner. Considering that vitamin B5 is a precursor for the synthesis of coenzyme A (CoA), a molecule needed to activate fatty acids for oxidative phosphorylation in mitochondria, the authors suggested that TDD may be a disorder of lipid metabolism. Accordingly, TANGO2 might help regulate the levels of various chain lengths of acyl-CoA conjugates, which are the key regulators of many metabolic enzymes participating in complex lipid synthesis, protein acylation and subcellular signaling pathways (Grevengoed et al., 2014).

**Fig. 3.**
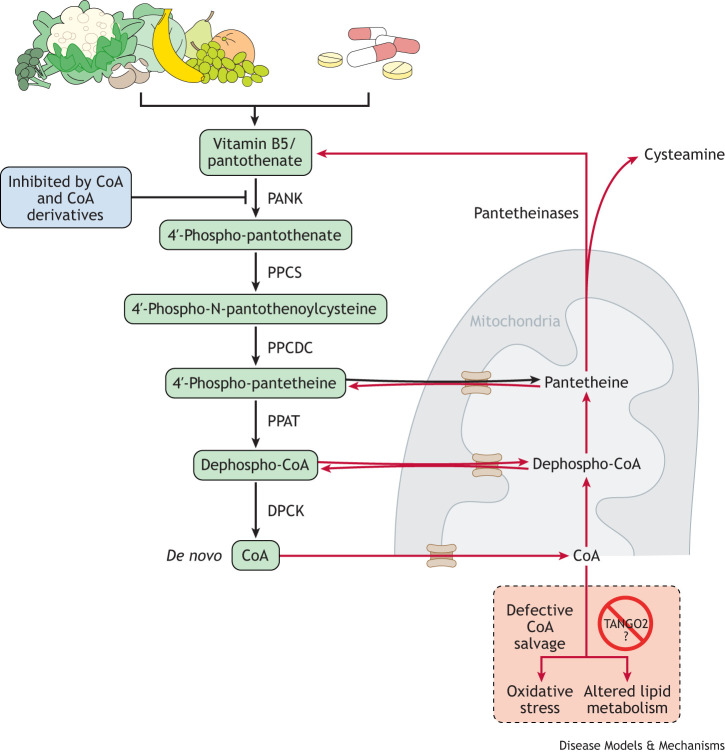
**Pathways of coenzyme A synthesis.** Pantothenate (vitamin B5), the obligate precursor of coenzyme A (CoA), is generally ingested either by food or vitamin supplements. A five-step process converts this vitamin into CoA. In order to regulate CoA levels, a balance between *de novo* synthesis and salvage versus degradation and synthesis inhibition must be achieved. Defects in salvage could lead to altered lipid metabolism and/or oxidative stress. CoA salvage is largely mediated by the levels of the antioxidant cysteamine, but a defect in CoA salvage in the absence of TANGO2 could help explain patient phenotypes and why vitamin B5 supplementation alleviates metabolic crises. Red arrows indicate processes involved in CoA salvage, with much of this process involving transport of metabolites into or out of the mitochondria. PANK, pantothenate kinase; PPCS, phosphopantothenoylcysteine synthetase; PPCDC, phosphopantothenoylcysteine decarboxylase; PPAT, phosphopantetheine adenyl transferase; DPCK, dephospho-CoA kinase.

To investigate how *TANGO2* depletion affects the lipidome, Malhotra and colleagues (Lujan et al., 2023) knocked down *TANGO2* in human hepatocytes (HepG2 cells). Their lipidomics analysis revealed decreased phospholipid levels, particularly phosphatidic acid, along with a simultaneous increase in the corresponding lysophospholipid, lysophosphatidic acid. The data suggest that TANGO2 promotes conversion of the lysophospholipids into phospholipids either directly through an enzymatic reaction of lipid acylation or indirectly by increasing cellular acyl-CoA to augment the process (Fig. 3). Moreover, TANGO2 depletion in this system led to a reduction in the levels of cardiolipins, which are important phospholipids found exclusively in the mitochondria (Horvath and Daum, 2013; Jiang et al., 2022; Mileykovskaya and Dowhan, 2009). Defects in mitochondrial morphology were previously reported in fibroblasts derived from individuals with TDD (Milev et al., 2021). There is also evidence suggesting that TANGO2 is localized to mitochondria (Milev et al., 2021) and partially to lipid droplets and the endoplasmic reticulum (Lujan et al., 2023). This highlights a possible role of TANGO2 in mitochondrial function and morphology, likely mediated through cardiolipins. Furthermore, TANGO2-depleted HepG2 cells showed increased levels of reactive oxygen species (ROS)-induced lipid peroxidation (Lujan et al., 2023). Pathological lipid peroxidation damages biomembranes and triggers programmed cell death via apoptosis, autophagy and ferroptosis (reviewed in Su et al., 2019). Phospholipid oxidation also promotes inflammation and disease. Interestingly, several B vitamins [e.g. vitamin B1 (thiamine), the vitamin B6 derivative pyridoxal, vitamin B12 and vitamin B5] can reduce oxidative stress (Depeint et al., 2006). These results suggest that providing TANGO2-deficient cells with nonenzymatic antioxidants, such as vitamins and their analogs, minerals, and metabolites (Su et al., 2019) may mitigate ROS activity, which calls for more research on their efficacy.

Future studies are warranted to further elucidate how different B vitamins, especially vitamin B5, can rescue TANGO2 deficiency-related defects in different human cell lines and model organisms.

Preliminary lipidomic analysis from our group (Mehranfar et al., 2024) revealed a significant increase in the abundance of unsaturated free fatty acids, neutral lipids, sphingomyelins, and phospholipids in primary human TANGO2-deficient fibroblasts harboring the exon 3-9 deletion commonly found in patients with TDD, compared to the levels seen in control cells. This increase was particularly notable for triglycerides, diacylglycerides and ceramides, which was further exacerbated during glucose starvation, suggesting a potential defect in lipid consumption when cells need fatty acids for energy release. Importantly, the levels of unsaturated fatty acids, triglycerides and diacylglycerides were rescued when cells were treated with vitamin B5. Furthermore, a recent global lipidomic analysis of *tango2* mutant zebrafish and control lines revealed an overall reduction of lipids, particularly phosphatidylcholine, phosphatidylethanolamine, lysophosphatidylcholine and triglyceride (Kim et al., 2023).

All these studies agree on a likely critical role for TANGO2 in lipid metabolism. Intriguingly, the specific lipid species being perturbed differ among model organisms. This could be due to TANGO2 having different, multiple and/or species-specific functions. Alternatively, TANGO2 may function upstream of lipid mobilization, with its effect simply manifesting differently in each system. Such differences may also reflect the properties of the model systems used and the method of TANGO2 depletion (RNA interference, natural knockout or CRISPR/Cas9-mediated knockout). Moreover, different extraction methodologies, technologies used and potential misidentifications all contribute to the resulting profiles and may yield discrepancies and non-reproducibility in lipidomic analyses. Improved resolution of modern mass spectrometry systems has made lipidomics research more efficient in analyzing complex biological matrices, overcoming technical and biological variability. Technical reproducibility and analytical precision, reflected by low relative standard deviation values, ensure the accuracy of lipid concentration measurements, which facilitates the identification of meaningful patterns and variations (Smirnov et al., 2021).

These recent findings highlight the importance of TANGO2 function in lipid homeostasis and aspects of acyl-CoA metabolism either directly or indirectly. Future studies are warranted to further elucidate how different B vitamins, especially vitamin B5, can rescue TANGO2 deficiency-related defects in different human cell lines and model organisms.

## Is TANGO2 a heme chaperone?

A recent paper by Sun et al. (2022) suggests that TANGO2 acts as a heme chaperone in multiple model systems including worms, flies, fish and human cells. As accumulating evidence suggests defective lipid homeostasis is the likely cause of TDD and no clinical features of individuals with TDD can be related to a heme-trafficking defect (Miyake et al., 2023), the models used by Sun et al. (2022) have been re-examined by several laboratories. Although the results in both the yeast and zebrafish models could not be replicated, new data suggest that the worm heme phenotype may be explained by reduced feeding in the knockout worms (Sandkuhler et al., 2023b preprint). More recently, a bacterial TANGO2 homolog was suggested to bind to heme through a critical conserved tyrosine residue (tyrosine 116 in the canonical human protein, Fig. 2) (Han et al., 2023). So, how can the heme reports be reconciled with the emerging lipid homeostasis data? It is noteworthy that no missense variants at tyrosine 116 have been reported in humans, yet two variants (histidine and cysteine) are found in gnomAD, neither in the homozygous state. TANGO2 may be a heme chaperone, but this function alone does not seem to contribute to the disease state. Alternatively, as TANGO2 likely interacts with non-polar lipids, an interaction with the hydrophobic heme molecule may occur non-specifically. Identifying and characterizing the clinical phenotype of individuals with tyrosine 116 missense variants (if any) will help resolve this controversy.

## Conclusion

Although the precise function of TANGO2 remains a mystery, accumulating evidence clearly indicates that TANGO2 mutation disrupts lipid homeostasis and causes ROS damage. Either multivitamins that include all eight B vitamins, vitamin B complex, or vitamin B5 only have been shown to prevent metabolic crises in patients with TDD, possibly due to their stimulation of coenzyme A and/or lipid biosynthesis and antioxidant properties (Miyake et al., 2023; Sandkuhler et al., 2023a). With proper dosage, vitamin supplementation is generally well tolerated and suitable to the long-term administration needed to manage chronic TDD. Considering that ROS damage and lipid peroxidation may trigger cell death via the mitochondria (Salami et al., 2019; Su et al., 2019; Villanueva et al., 2013), vitamin B5, possibly supplemented with other antioxidant B vitamins and minerals, may counter these effects and rescue the disrupted energy metabolism in TDD. Distinct *TANGO2* alleles and allele combinations may respond differently to treatment, depending on the molecular properties of the corresponding mutant protein. Therefore, although a vitamin B5 or vitamin B complex course relieves critical symptoms in the short term, model organism studies will be key to determining the cellular function(s) of TANGO2 in cardiac and other tissues and to find out which ones participate in the crises directly or indirectly.
